# The Role of Defending Norms in Victims’ Classroom Climate Perceptions and Psychosocial Maladjustment in Secondary School

**DOI:** 10.1007/s10802-020-00738-0

**Published:** 2020-12-10

**Authors:** Lydia Laninga-Wijnen, Yvonne H. M. van den Berg, Tim Mainhard, Antonius H. N. Cillessen

**Affiliations:** 1 Interdisciplinary Social Science, Utrecht University, University of Groningen, Sociology, Padualaan 14, 3584 CS Utrecht, The Netherlands; 2 Behavioural Science Institute, Radboud University, Nijmegen, The Netherlands; 3 Department of Education, Utrecht University, Utrecht, The Netherlands; 4 Behavioural Science Institute, Radboud University, Nijmegen, The Netherlands

**Keywords:** Popularity norms, Descriptive norms, Defending, Classroom climate, Social-emotional adjustment, Victimization

## Abstract

Victims of bullying are at increased risk of developing psychosocial problems. It is often claimed that it helps victims when others stand up against the bullying and when defending is typical (descriptive norm) or rewarded with popularity (popularity norm) in classrooms. However, recent work on the healthy context paradox suggests that victims – paradoxically – tend to do *worse* in more positive classrooms. Therefore, it is possible that defending norms are counterproductive and exacerbate victims’ adjustment difficulties, possibly because social maladjustment is more apparent in classrooms where everybody else is doing well. The current study examined whether descriptive and popularity norms for defending predicted victims’ classroom climate perceptions and psychosocial adjustment. Using data of 1,206 secondary school students from 45 classrooms (*M*_age_ = 13.61), multi-level analyses indicated that descriptive norms for defending increased rather than decreased negative classroom climate perceptions and maladjustment of victimized youths. In contrast, popularity norms for defending positively predicted all students’ classroom climate perceptions and feelings of belonging, except victims’ self-esteem. Interventions may benefit more from promoting popularity norms for defending rather than descriptive norms for defending in secondary schools.

In adolescence, when the desire for inclusion and acceptance by peers is high, being victimized by peers takes a significant social-emotional toll. Being victimized signals to youths that they are not welcome in their peer group. This experience may lead to negative classroom climate perceptions and maladjustment, such as low self-esteem and loneliness (Arsenault 2018). Nonetheless, stress-buffer theories of social support (Cohen and Wills 1985) posit that such maladaptive associations can be attenuated when victims also feel supported by their peers, such as when they are defended by classmates (Sainio et al. 2011). Defending is the process in which peers comfort and support victims, stand up against a bully, or seek help from adults (Reijntjes et al. 2016). Being defended may make victims feel supported, less alone, and better able to cope with their situation which is reflected in their classroom climate perceptions and psychosocial functioning (Schacter and Juvonen 2019).

Not surprisingly, promoting the defending of victims has been the focus of anti-bullying programs (see, for overviews, Evans et al. 2014; Yeager et al. 2018). Some interventions aim at generating *defending norms* in classrooms so that bullying becomes disapproved and defending is approved and accepted. In the literature, two types of norms are often distinguished. Descriptive norms refer to the average level of perceived defending in the classroom. Popularity norms refer to the degree to which defending is associated with popularity in the classroom (cf. Henry et al. 2000; Laninga-Wijnen et al. 2020a).

Stress-buffer theories (Cohen and Wills 1985) argue that victims benefit from defending norms. But recent research has shown an opposite pattern, referred to as the *healthy context paradox* (Huitsing et al. 2019). The paradox is that the adjustment difficulties of victimized youth were actually larger, not smaller, in healthier classrooms in these studies. For example, among 10- to 12-year-olds, victims had lower self-esteem and more depressive symptoms in classrooms with lower victimization rates (Huitsing et al. 2012). In another study, victimized adolescents experienced more somatic problems in classrooms with lower victimization rates (Gini et al. 2020). In a daily diary study, the impact of verbal victimization on children’s negative self-views was larger in classrooms with less aggression (Morrow et al. 2019). Thus, victims in less negative classrooms did worse than victims in more negative classrooms.

It is unclear whether the healthy context paradox also holds for the presence of positive classroom aspects, such as defending norms. There are only a few studies on defending norms. They almost exclusively focused on descriptive norms for defending, not popularity norms, and on how these norms affect defenders, not victims (Kubiszewski et al. 2019; Troop-Gordon et al. 2019; Yun and Graham 2018)*.* These studies showed that descriptive norms for defending increased students’ willingness to defend victims. But it remains unclear how defending norms actually affect the victims that they are intended for. Moreover, making the distinction between defending popularity norms and descriptive norms is important, particularly in adolescence when the desire for popularity steadily increases (LaFontana and Cillessen 2010). The behaviors displayed by popular peers are likely to be seen in a positive light and may present a more powerful norm to classmates than the behaviors that are generally displayed by *all* peers. Indeed, popularity norms rather than descriptive norms were found to play an important role in adolescents’ school life (Dijkstra and Gest 2015; Laninga-Wijnen et al. 2020a). Consequently, the goal of this study was to examine how descriptive and popularity norms for defending are related to perceptions of classroom climate and psychosocial adjustment (self-esteem, belonging, loneliness) of secondary school students who are victimized by their peers. We considered four classroom climate indicators (cf. Boor-Klip et al. 2016), assessing the extent to which students perceive a positive, inclusive group structure (cohesion), and a lack of exclusion (isolation), as well as high degrees of helping (cooperation) and a lack of conflictive, negative behaviors (conflict). Consequently, both interactional and relational aspects of the classroom climate were captured (Rubin et al. 2006).

## Defending Descriptive Norms

The healthy context paradox (Huitsing et al. 2019) states that strong defending descriptive norms work adversely for victims for three reasons. First, being victimized in an otherwise positive classroom may negatively impact victims’ causal attributions of their situation. Victims make attributions to explain why they are victimized (Graham and Juvonen 1998). These attributions include locus (whether the cause of is internal or external to the victim) and controllability (whether the cause can be changed). In classrooms where defending behaviors are widespread, it is likely that victimization is uncommon (Saarento et al. 2015) and being a victim of bullying is thus not normative. Consequently, victims are more likely to blame themselves (i.e., make an internal attribution) as they are the only one or one of the few who have this problem. They may also conclude that nothing can be done about it (uncontrollable), because the many defending efforts in the classrooms did not to stop the perpetrators (Kaufman et al. 2020). Victims who make internal and uncontrollable attributions have more psychosocial problems than victims who do not make these attributions (Schacter et al. 2015). Thus, defending descriptive norms may relate to more negative classroom climate perceptions and psychosocial maladjustment in victims.

Second, social comparison theory (Festinger 1954) states that people generally compare their situation with that of others to define their self-worth. In classrooms with strong defending descriptive norms, victims mainly have “upward social comparison material”: most peers are better off. These peers are seen to be able to defend victims against bullying and to have the resources to offer help (e.g., being confident about oneself, or being surrounded by supportive friends who can shield from a revenge of the bully; Hawley 2014). Having many classmates with such resources yields more opportunity for upward comparisons. These comparisons in turn emphasize victims’ own disadvantaged position, making them feel even worse about themselves and their situation in a in generally positive classroom.

Third, victims may perceive defending as less effective in classrooms where it is widespread (a so-called *defending-inflation*). Defending may be *less* effective in classrooms with high defending descriptive norms because in such classrooms, *all* students may try to defend a victim including those who may be unsuitable for it, such as unpopular or other victimized peers. Indeed, being helped by someone who is victimized as well may exacerbate victims’ problems (Guarneri-White et al. 2015; Schacter and Juvonen 2020). Therefore, defending in classrooms with high defending descriptive norms may be inefficient to stop the bully and instead escalate psychosocial problems and negative feelings about oneself and the classroom. Based on these arguments, we hypothesized that defending descriptive norms may amplify the association between victimization and negative classroom climate perceptions and psychosocial maladjustment (Hypothesis 1).

## Defending Popularity Norms

Are popularity norms also subject to the healthy context paradox? Contrasting hypotheses are possible. On the one hand, defending popularity norms – just like descriptive norms – may exacerbate the link between victimization and maladjustment or negative classroom experiences. This may again be due to the negative attributions victims may develop in such classrooms, or because the popular defending behaviors are not sufficiently effective for victims. Moreover, when defending is related to popularity, students may primarily defend to gain status for themselves (self-serving goal) rather than to help the victim (other-oriented goals). Indeed, defending can be motivated by personal gains, such as status gains – in particular when opposing a bully (Pronk et al. 2019). Importantly, the goals that underlie prosocial behavior may affect the quality of the help that is provided. For example, people with self-serving goals seem more likely to give dependency-enhancing help (Halabi et al. 2008; Jackson and Esses 2000). If this occurs in defending situations, victims may become dependent on their defender and may feel inferior and powerless as it may strengthen the idea that they are unable themselves to stand up against the bully. It can thus be hypothesized that defending popularity norms amplify the link between victimization, negative classroom climate perceptions, and psychosocial maladjustment (Hypothesis 2A).

On the other hand, defending popularity norms may *not* be susceptible to the healthy context paradox and actually *decrease* the adverse effects of victimization. This could work in two ways. First, as adolescents seek popularity, behaviors associated with popularity are valued (Dijkstra and Gest 2015). These behaviors become reputationally salient: a valuable tool to gain popularity in the peer group (Hartup 1996). Consequently, students approve defending behaviors and are unlikely to stand up against them (Henry et al. 2000; Laninga-Wijnen et al. 2020a). It will be comforting for the defenders (and the victims) to know that their peers have their back when they stand up against a bully, as defending can be risky (Garandeau et al. 2018). When popularity norms for defending are stronger, it is less likely to be disputed and it more effectively signals that people *do* care about the victim. This will be advantageous for victims’ adjustment and classroom climate perceptions.

There may be a second way in which popularity norms work positively for victims. High defending popularity norms imply that popular peers engage in defending and unpopular peers do not. Being defended by an unpopular (or) victimized peers may not be effective or exacerbate problems (Guarneri-White et al. 2015; Schacter and Juvonen 2020). But popular defenders have the social power and impact to deter bullies. This power may be indispensable, as bullies are often popular themselves (Garandeau et al. 2018) and look down on lower-status peers (Van Kleef et al. 2008). Popular defenders may decrease the status of bullies by standing up against them (Laninga-Wijnen et al. 2020a). Consequently, in classrooms with high defending popularity norms, victims may develop a cognitive anticipation and trust that the bullying will cease over time. Based on this reasoning, a contrasting hypothesis is that defending popularity norms will *not* be subject to the healthy context paradox but instead work out positively for victims’ classroom climate perceptions and adjustment (Hypothesis 2B).

## Present Study

The aim of this study was to clarify the role of descriptive and popularity norms for defending in the psychosocial adjustment and classroom climate perceptions of victimized youths. In line with the healthy context paradox, we expected that victims in classrooms with high descriptive norms for defending are worse off than victims in classrooms with low descriptive norms for defending (Hypothesis 1). We had two contrasting hypotheses for popularity norms. On the one hand, popularity norms may be susceptible to the healthy context paradox in similar ways as descriptive norms, hence enhance the plight of victims (Hypothesis 2A). On the other hand, victims may be better off in classrooms with high popularity norms than in classrooms with low popularity norms (Hypothesis 2B), because popular defenders may have the power to deter bullies and because defending is less likely to be disputed by others.

We tested our hypotheses for defending norms while controlling for classroom-levels of victimization (Dijkstra and Gest 2015). In this way, we extended prior work by disentangling the absence of negative aspects (e.g., low victimization) from the presence of positive aspects (defending norms). Based on prior work (Huitsing et al. 2012), we expected victims to do better in classrooms with higher average levels of victimization (Hypothesis 3).

Classroom-level factors may not operate in isolation to affect victims’ classroom climate perceptions and adjustment. Strong defending descriptive norms may be particularly harmful for victims when classroom-levels of victimization are low. At the same time, it may help victims to be in classrooms where defending is effectively shown by popular peers (popularity norm) *and* where they are not the only victim. Furthermore, a context characterized by both descriptive *and* popularity norms for defending may work out positively, because the defending is widespread *and* associated with popularity. Being in a classroom without any norm of defending may be indicative of no effort to stop the bullying or to make a victim feel better, which may work out negatively for victims’ classroom climate perceptions and adjustment. Therefore, in additional analyses, the interactions between classroom levels of victimization and norms were explored (i.e., descriptive or popularity norms).

We conducted our study in a sample of secondary school students, which adds to the literature in several ways. Most prior work on the role of classroom factors in victims’ adjustment focused on elementary school students. In the Netherlands, the classroom also constitutes an important context for secondary students, given that they spend most or even all of their time within the same classroom peers across an entire school year. Examining classroom norms, victimization, and adjustment in secondary schools clarifies whether the theoretical rationale for younger ages also applies at later ages. Victimization may occur more indirectly and may be less visible for teachers in secondary school (students have up to 15 teachers for varying subjects); this may enhance the adverse impact of victimization and therefore leaves less room for contextual factors to buffer against this. Moreover, compared to children, adolescents attach increasing value to peer popularity (Li and Wright 2014), hence, parsing out the relative impact of popularity norms versus descriptive norms is key to understand the role of the broader classroom context in this developmental period.

## Method

### Procedure

Data were retrieved from the Kandinsky Longitudinal Study, which started in 2010, initiated by the head of a large secondary school who requested a yearly assessment of the social-emotional adjustment of students. The school head requested parental permission at the beginning of each school year for all studies that the school considered to be necessary for students’ well-being. The school signed a letter in which they formally requested the research team to monitor socio-emotional well-being of their students and in which they claimed the responsibility for the parental consent procedure. The school distributed a letter to parents which described the purpose and procedures of the study, including the option to exclude their child(ren) from participation. None of the parents objected participation of their children. Prior to testing, participants were verbally informed about the goal of the study and confidentiality of answers was emphasized. Participants could opt out any time. Active informed assent was requested at the start of each assessment; none of the adolescents objected participation.

Data collection took place in November and December. During the assessment (45–60 min classroom session), talking was prohibited to guarantee participants’ privacy and assessment reliability. All participants sat in a test arrangement with adequate space between private desks. Dividers were placed around the computer screens, avoiding that students would see each other’s computer screen. There were always at least two researchers present during data collection to make sure instructions were followed and to answer any questions students had. Procedures are in line with ethical guidelines for sociometric research (Guideline 2, Bell-Dolan and Wessler 1994), in agreement with school policies, and approved by the Institutional Review Board of our university (Radboud University of Nijmegen, The Netherlands).

### Participants

For the current study, we analyzed data from Wave 7 (2016) specifically as students were asked about their classroom climate perceptions in this particular wave. All adolescents in Grades 7^th^ to 10^th^ were assessed. These are students in the first four years of secondary education in The Netherlands. A total of *N* = 1,310 adolescents from 49 classrooms participated. All 7^th^ to 9^th^ grade students (81.4% of our sample) followed the same classes with the same classmates throughout the school year; thus they remained within the same group of students every hour, every day. The 9^th^ grade students sometimes followed an additional class next to their basic curriculum, which could partly take place in another classroom with students in the same grade that were not classmates; yet this only took at most a few hours a week. The 10^th^ grade students had some classes with students from their grade other than their classmates, but still spent the majority of their time with their classmates as they followed all core courses and mentor hours together.

**Missingness.** Out of the 1,310 participants, 79 students were absent when the questionnaire was administered. Due to time restrictions, some students had missing scores on the classroom climate perceptions items, which were administered at the end of the questionnaire. In total, 34.6% of the first-year students did not fill in the questionnaire about classroom climate perceptions, compared to 18.6%-21.3% of the second-to fourth year students, *X*^2^(3) = 33.56, *p* < 0.001, *φ * = 0.16. In order to address this missingness, we excluded four classrooms for which we had information of less than 10 students on the classroom climate data (Garandeau et al. in press). This resulted in a final sample of 1,206 students from 45 classrooms. Little’s missing completely at random test produced a normed chi-square (*X*^2^/*df*) of 1.44, indicating that it was safe to impute missing values (Bollen 1989). We estimated missing values for our variables of interest using the Expectation Maximization procedure, with all study measures as predictors (Groothuis-Oudshoorn & van Buuren, 2011, p. 22 ; Gupta & Chen, 2010).

Of the 1,206 students, 49.0% were female. The distribution across grades was 36.1% in Grade 7 (*n* = 435, *M*_age_ = 12.61, *SD* = 0.44), 22.1% in Grade 8 (*n* = 267, *M*_age_ = 13.59, *SD* = 0.43), 23.2% in Grade 9 (*n* = 280, *M*_age_ = 14.61, *SD* = 0.43), and 18.6% in Grade 10 (*n* = 224, *M*_age_ = 15.96, *SD* = 0.68). Most adolescents (90.0%) indicated that they were born in the Netherlands.

## Measures

### Individual-level Predictor Variables

Victimization was measured using a revised version of the Olweus questionnaire (Olweus 1996), consisting of six questions about victimization experiences. This included the extent to which others spread rumors about a person (1), pushed, kicked, or hit a person (2), called names (3), bullied (4), ignored or excluded (5), or sent hurtful messages through the internet (6). Adolescents indicated on a five-point Likert scale how often they were victimized in each of these ways since the beginning of the academic year (1 = never, 2 = 1 or 2 times, 3 = 1 to 3 times a month, 4 = 1–2 times a week, 5 = 3 or more times a week). Exploratory factor analysis in M*plus* indicated that the six items loaded strongly (geomin rotated factor loadings > 0.52) on one factor with an eigenvalue of 2.95, accompanied by a proper model fit [RMSEA = 0.07, CFI = 0.98, TLI = 0.96, SRMR = 0.02]. Cronbach’s *a* was good (α = 0.78).

**Gender and Age.** Boys were coded 0 and girls were coded 1. We included the classroom-mean centered age as covariate (range = 11–18 years) in the analyses.

### Individual-level Outcome Variables

**Classroom Climate Perceptions.** We used four subscales of the Classroom Peer Context Questionnaire (Boor-Klip et al. 2016): cooperation (4 items), conflict (4 items), cohesion (3 items), and isolation (4 items). Example items were: “In this classroom,”… “youths help each other” (cooperation), “youths argue with each other” (conflict), “everyone plays together in the break” (cohesion), and “some youth do not belong to the group” (isolation). Adolescents rated each item on a 5-point Likert scale (1 = not true at all; 5 = completely true). The average score was calculated for each scale. Higher scores indicated more positive classroom climate perceptions, thus *lower* levels of isolation and conflict, and *higher levels* of cooperation and cohesion. Cronbach’s α was good for all scales (0.84, 0.80, 0.75, and 0.77 for cooperation, conflict, cohesion, and isolation, respectively).

**Feelings of Belonging.** The subscale “comfort” of the CPCQ was used to measure students’ feelings of belonging to the classroom (4 items; Boor-Klip et al. 2016). An example item was: “In this classroom, I can be myself”. These items were averaged, with higher scores indicating stronger feelings of belonging. Cronbach’s alpha was good (α = 0.87). We conducted confirmatory factor analyses to examine whether the five-factor structure of the CPCQ (cooperation, cohesion, isolation, conflict, and feelings of belonging) held in our secondary school sample, which was the case [RMSEA = 0.06, CFI = 0.92, TLI = 0.91, SRMR = 0.07].

**Self-esteem.** Self-esteem was measured with the 10-item Rosenberg Self-Esteem Scale (RSS; Rosenberg 1965). Adolescents rated each item on a 5-point Likert scale (1 = not at all; 4 = very much). All items were averaged, with higher scores being indicative of higher self-esteem. Cronbach’s alpha was good (α = 0.88).

**Loneliness**. The Loneliness and Aloneness Scale for Children and Adolescents (LACA; Marcoen et al. 1987) consisted of 12 items that rated answered on a 4-point Likert-scale (1 = never, 4 = often). Higher scores indicate more loneliness in the peer context. An example item was “I feel alone at school”. Cronbach’s alpha was good (α = 0.88). Item scores were averaged. Yet, the distribution of this score was skewed and peaked (skewness = 1.87, kurtosis = 4.38). Therefore, we recoded the scale into three categories (1 – 1.5 = 1, 1.5 – 2 = 2, > 2 = 3).

### Classroom-level Predictor Variables

We measured classroom norms with peer nominations. Each nomination question was presented on top of a separate screen, followed by the names of all classmates. Participants could name as many or as few classmates as they wanted for each question but not themselves.

**Defending Descriptive Norms.** Descriptive norms were operationalized as the classroom-level average of peer-nominated defending (“Who defends classmates who are victimized?”). All adolescents received an indegree-score, indicating the number of classmates that nominated them for this question. To control for classroom size, the number of nominations received was divided by the number of nominators in the classroom to a proportion score. These proportion scores were aggregated at the classroom-level and *z-*standardized across all classrooms to create a measure of descriptive norms for defending.

**Defending Popularity Norms.** Defending popularity norms were calculated as the within-classroom correlation between peer-nominated defending and peer-nominated popularity. Popularity was assessed by asking participants to name classmates who were most popular and least popular. Nominations received were counted for each participant for each question and divided by the number of nominators in each classroom to create proportion scores. The proportion score for least popular was subtracted from the proportion score for most popular to a final score for popularity (e.g., Lease et al. 2002). For each classroom, the correlation between popularity and defending was calculated (cf. at least 12 other studies examining popularity norms; e.g., Dijkstra and Gest 2015; Laninga-Wijnen et al. 2020a). These scores were transformed to Fisher *z*-scores using the formula: 0.5*[*ln*(1 + *r*)-*ln*(1-*r*)] (Fisher 1925; cf. Laninga-Wijnen et al. 2020a) in order to obtain a normally distributed measure.

**Average Victimization Levels in Classrooms.** We controlled for the average level of victimization in classrooms by aggregating children’s average score on the Revised Olweus Questionnaire at the classroom-level (Olweus 1996).

**Grade.** We entered grade level as control variable in the analyses.

## Analyses

We conducted multi-level regression analyses in M*plus* Version 8 (Muthén and Muthén 2016) to take the nested structure of the data into account. Classroom-level variables were grand-mean centered; individual-level variables were centered at the classroom mean. We used the MLR-estimator (Yuan and Bentler 2000) to account for the potential non-normal distribution of the residuals. We tested one model for each outcome separately (cohesion, cooperation, isolation, conflict; belonging, loneliness, self-esteem), as including all outcomes simultaneously resulted in too many parameters and model nonconvergence.

The analyses included four steps. First, we tested empty models and examined intraclass correlations (ICC). Second, we included the individual- and classroom-level predictors to explain variance in the outcome variable at these two levels (Main models, Model A.1, Appendix 1). These main models were used to assess the role of individual-level and classroom-level variables on our outcome variables of interest. Third, we included the random slope for the association between individual victimization and the outcome variable, to see whether there were statistically significant differences between classrooms in the effect of students’ victimization (Model B.1). Fourth, we simultaneously included three two-way cross-level interactions (i.e., defending popularity norm*individual victimization, defending descriptive norm*individual victimization, and classroom-level victimization*individual victimization) to test whether the variability in the association between students’ victimization and classroom climate perceptions or school adjustment was explained by the defending descriptive norms, popularity norms, and average levels of victimization in classrooms (Model C.1). We included these three two-way cross-level interactions *simultaneously,* to account for potential confounding and to parse out the relative effects of descriptive norms, defending norms, and victimization levels. We interpreted cross-level interactions only if 1) models containing these interactions (Models C.1) had a better fit (lower AIC) compared to Model A.1; and 2) at least one of the cross-level interactions was significant and predicted a relatively large part of the variance in the random slope (≥ 20%; Cohen 1988). We conducted simple slopes analyses with the Preacher and Hayes method for multi-level analyses (Preacher et al. 2006). A score of -1 was entered in the formula to indicate low victimization on the standardized victimization scale (thus referring to those scoring 1 SD below the mean of victimization). We also calculated coefficients for students who were moderate (0), high (1), and very high (2) on victimization. For descriptive norms, a score of -1 was entered in the formula to represent low norms (scoring 1 SD below standardized descriptive norms), whereas moderate norms received a '0’ and high norms received a ‘1’. For popularity norms, the value of ‘-0.6’ was used to refer to low norms, whereas values of '0’ and ‘0.6’ was used to refer to moderate and high norms, respectively (based on 95% interval of popularity norm scores).

## Results

### Descriptive Statistics

Table 1 presents descriptive statistics. Higher individual-level victimization correlated with more negative classroom climate perceptions (*more* perceived isolation and conflict, *less* cohesion and cooperation), lower self-esteem, lower feelings of belonging, and higher levels of loneliness. These correlations were significant and moderate-to-high in size. There were no statistically significant classroom-level correlations between defending popularity norms, defending descriptive norms, and classroom-levels of victimization. Higher grade levels were characterized by lower defending descriptive norms and lower victimization. Older adolescents were less likely to be victimized and perceived more cooperation and less conflict.

**Table 1:** Correlations between victimization, classroom climate perceptions, self-esteem, and loneliness, and classroom correlations

Individual-level	M(SD)	Range	1	2	3	4	5	6	7	8
1. Victimization (standardized)	0.00(1.00)	-.68; 7.81								
2. Cooperation	3.78(.56)	1.00; 5.00	-.27***							
3. Conflict	3.90(.64)	1.00; 5.00	-.49***	.43***						
4. Cohesion	3.07(.70)	1.00; 5.00	-.24***	.56***	.40***					
5. Isolation	3.35(.65)	1.25; 5.00	-.28***	.27***	.54***	.44***				
6. Belonging	4.07(.65)	1.00; 5.00	-.33***	.70***	.39***	.48***	.24***			
7. Self-esteem	3.19(.50)	1.10; 4.00	-.33***	.26***	.22***	.13***	.16***	.40***		
8. Loneliness	1.29(.59)	1.00; 3.00	.44***	-.28***	-.29***	-.23***	-.19***	-.47***	-.42***	
9. Age	13.91(1.33)	12.16; 16.37	-.10***	-.10**	.11***	.02	.05	-.21***	. 02	-.02
Classroom-level	M(SD)	Range	1	2	3					
1. Defending descriptive norm (standardized)	0.00(1.00)	-1.41; 2.48								
2. Defending popularity norm (Fisher z-score)	.08(.33)	-.65; .86	. 17							
3. Classroom average victimization	7.91(.77)	6.17; 9.66	-.03	-.22						
4. Grade	2.24(1.13)	1.00; 4.00	-.45**	.09	-.42**					

When interpreting scores and correlations for, mind that higher scores on conflict and isolation indicate *low levels* of perceived conflict or isolation (e.g., more positive classroom climate perceptions). For age, we reported the 5% and 95% percentiles.

### Intraclass Correlations

Classroom-level variation (ICC) was considerable for the classroom-related variables cooperation (0.14), cohesion (0.13), conflict (0.18), and isolation (0.14). It was moderate for feelings of belonging (0.09) and small for self-esteem (0.01) and loneliness (0.01).

### Individual-Level and Classroom-Level Predictors of Adolescent Adjustment

Before presenting the findings for our hypotheses, we first discuss the main effects of individual- and classroom-level characteristics on the outcomes in general. We interpreted main effects from models without cross-level interactions (Tables 2A and 3A).

**Table 2:** Main models and models with cross-level interactions predicting students’ perceptions of cooperation, conflict, isolation, and cohesion within the classroom

	Cohesion		Cooperation		Conflict		Isolation	
	A. Main models	B. Interactions	A. Main models	B. Interactions	A. Main models	B. Interactions	A. Main models	B. Interactions
Individual-level predictors								
Gender	-.02(.04)	-.02(.04)	.004(.03)	.01(.03)	.11(.03)**	.11(.03)**	-.07(.04)*	-.07(.04)*
Age	.03(.04)	.03(.04)	-.01(.04)	-.01(.04)	.02(.04)	.01(.04)	.06(.04)^+^	.06(.04)
Victimization	-.14(.02)***	-.17(.02)***	-.14(.02)***	-.15(.02)***	-.27(.02)***	-.28(.02)***	-.14(.02)***	-.16(.02)***
Classroom-level predictors								
Grade	-.03(.04)	-.03(.04)	-.06(.03)*	-.06(.03)^+^	-.02(.03)	-.02(.03)	-.07(.03)*	-.07(.03)*
Defending descriptive norm	.02(.03)	.02(.03)	.04(.03)	.04(.03)	-.01(.03)	-.01(.03)	-.01(.03)	-.01(.03)
Defending popularity norm	.32(.10)**	.32(.10)**	.23(.09)**	.23(.09)*	.25(.08)**	.25(.08)**	.20(.08)*	.20(.08)*
Classroom-level victimization	-.15(.05)**	-.15(.05)**	-.12(.04)**	-.11(.04)**	-.28(.04)***	-.28(.04)***	-.27(.04)**	-.27(.04)***
Two-way cross-level interactions								
Victimization*descriptive norm	-	-.04(.02)*	-	-.05(.02)*	-	.03(.03)	-	-.001(.02)
Victimization*popularity norm	-	.05(.08)	-	.03(.07)	-	.06(.06)	-	-.02(.08)
Victimization*classroom-level victimization	-	.06(.03)^+^	-	.03(.03)	-	.04(.03)	-	.04(.03)
Residual variances								
Residual variance within	.38(.02)***	.37(.02)***	.25(.02)***	.25(.02)***	.27(.02)***	.26(.02)***	.34(.02)***	.34(.02)***
Residual variance between	.03(.01)**	.03(.01)**	.02(.01)**	.02(.01)**	.01(.01)*	.02(.01)**	.02(.01)**	.02(.01)**
Variance explained								
Variance explained within	.05(.01)**		.07(.02)***		.21(.03)***		.05(.02)***	
Variance explained between	.53(.14)***		.51(.14)***		.81(.06)***		.74(.10)***	
Total % variance explained in outcome	10.5%		12.7%		31.3%		15.2%	

High scores on the conflict and isolation scale indicate *low* levels of conflict and isolation (thus, more positive classroom climate perceptions)

^+^*p* < .10; * *p* < .05; ** *p* < *.*01; *** *p* < .001

**Table 3:** Main models and models with cross-level interactions predicting students’ self-esteem, loneliness, and feelings of belonging

	Self-esteem		Loneliness		Feelings of belonging	
	A. Main models	B. Interactions	A. Main models	B. Interactions	A. Main models	B. Interactions
Individual-level predictors						
Gender	-.26(.03)***	-.25(.03)***	.64(.16)***	.64(.16)***	-.12(.03)***	-.12(.03)***
Age	.02(.03)	.02(.03)	-.07(.17)	-.07(.17)	-.002(.05)	.001(.05)
Victimization	-.18(.02)***	-.18(.02)***	.97(.09)***	1.07(.12)***	-.23(.02)***	-.24(.02)***
Classroom-level predictors						
Grade	.02(.02)	.02(.02)	.19(.11)^+^	.18(.11)	-.14(.02)***	-.14(.02)***
Defending descriptive norm	.03(.02)^+^	.04(.02)*	.07(.12)	.05(.12)	.04(.03)	.04(.03)
Defending popularity norm	-.003(.05)	-.001(.05)	-.18(.31)	-.20(.33)	.15(.07)*	.15(.07)*
Classroom-level victimization	-.03(.03)	-.03(.03)	.30(.14)*	.33(.15)*	-.12(.03)***	-.12(.03)***
Two-way cross-level interactions						
Victimization*descriptive norm	-	.01(.02)	-	.09(.12)	-	-.04(.02)*
Victimization*popularity norm	-	-.10(.04)*	-	.09(.27)	-	.10(.07)
Victimization*classroom-level victimization	-	-.01(.02)	-	-.15(.10)	-	.01(.02)
Residual variances						
Residual variance within	.21(.01)***	.20(.01)***	n.a	n.a	.33(.02)***	.33(.02)***
Residual variance between	.004(.002)^+^	.004(.002)^+^	.10(.07)	.10(.07)	.01(.004)*	.010(.004)*
Variance explained						
Variance explained within	.17(.02)***		.22(.04)***		.13(.02)***	
Variance explained between	.38(.21)^+^		.34(.22)		.79(.10)***	
Total % variance explained in outcome	17.6%		22.4%		18.0%	

This model has been conducted using logistic multi-level analyses. n.a. = not available for logistic regression

^+^ *p* < .10; * *p* < .05; ** *p* < *.*01; *** *p* < .001

**Individual-level Predictors.** Greater victimization related to more negative classroom climate perceptions, lower self-esteem, lower feelings of belonging, and more loneliness. Girls perceived less conflict but more isolation than boys, and had lower self-esteem, lower feelings of belonging, and more loneliness. No age effects were found. Across outcomes, the individual-level variables explained 4.5% to 22.0% of the variance at the individual level.

**Classroom-level Predictors.** Defending descriptive norms were unrelated to classroom climate perceptions and psychosocial adjustment. Higher defending popularity norms related to stronger feelings of belonging and more positive perceptions of cooperation and cohesion at the classroom level. Positive *B-*coefficients indicated that higher defending popularity norms related to more positive perceptions of isolation and conflict at classroom level as well (i.e., *lower* isolation and conflict). Greater classroom-level victimization related to lower feelings of belonging and more negative classroom climate perceptions (more isolation and conflict, less cooperation and cohesion). Classroom-level victimization also related to greater loneliness, but given the very low ICC of loneliness and the non-significant *R*^*2*^*,* this effect is negligible. In higher grades, students experienced more isolation and less belonging. Across outcomes, classroom-level predictors explained 34.0% to 80.7% of the classroom-level variance.

### Defending Descriptive Norms and Victims’ Adjustment and Classroom Perceptions

In order to examine the role of defending descriptive norms in victims’ psychosocial adjustment and classroom perceptions, we analyzed the cross-level interactions (Table 2B and 3B). Three of the seven were significant, which supported Hypothesis 1 that adolescent victims are *worse off* in classrooms with stronger defending descriptive norms.

**Fig. 1:**
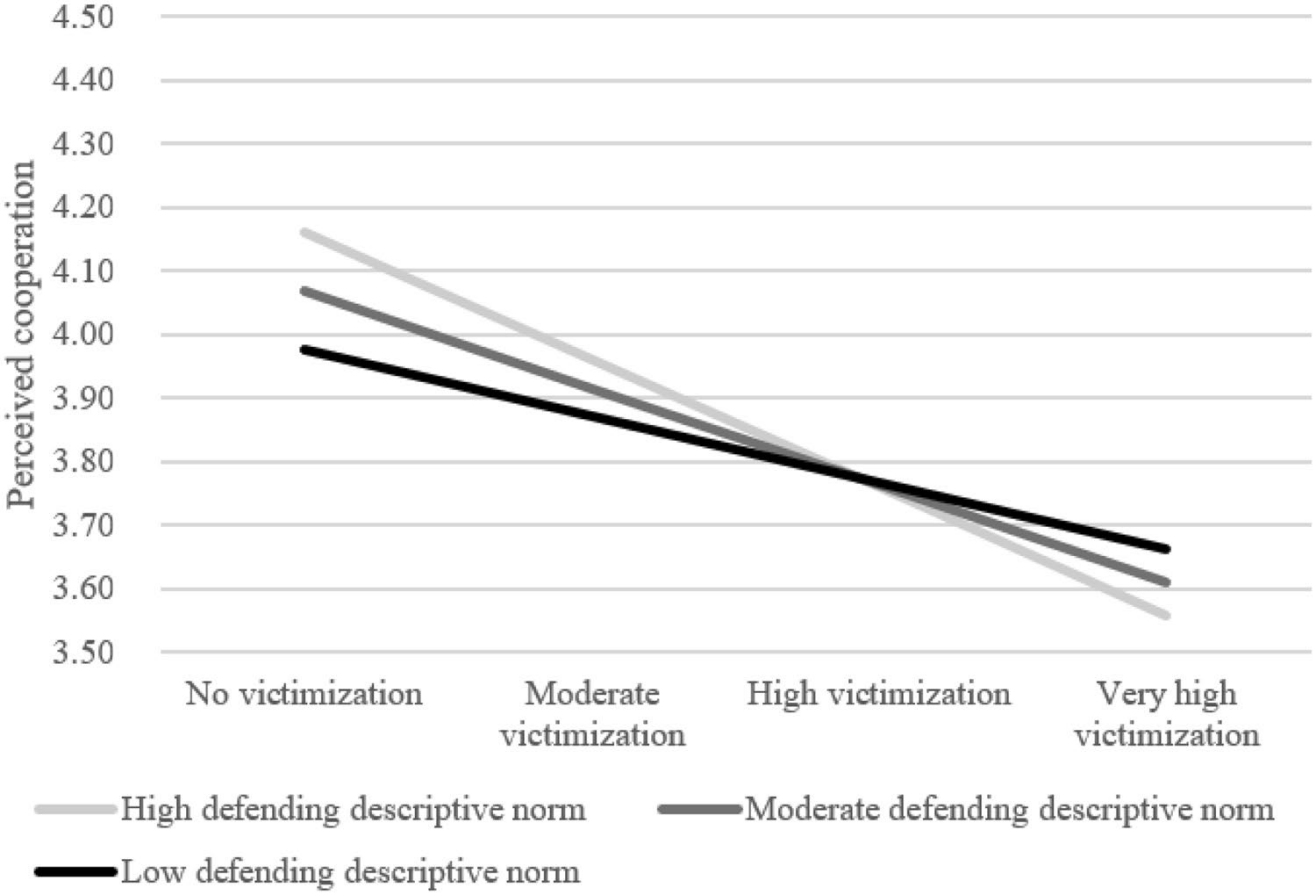
The role of defending descriptive norms in the link between victimization and perceptions of cooperation within classrooms. High defending norms are > 1 SD above the mean whereas low defending norms are > 1 SD under the mean. Low victimization = score of -1 on standardized victimization scale. Moderate victimization = score of 0, high victimization = a score of 1, and very high victimization = 2

First, the random slope of victimization on perceived cooperation was significantly predicted by descriptive norms, *B* = -0.05, *SE* = 0.02, *p* = 0.025, explaining 20.0% of the variation of the slope across classrooms (Model 1.C.1 vs. Model 1.B.1, Appendix 1). Figure 1 indicates that the negative association between victimization and perceptions of cooperation was stronger when defending descriptive norms were higher. Specifically, descriptive norms were positively related to perceptions of cooperation for non-victimized youths. They were unrelated to perceptions of cooperation for youth at moderate-to-high levels of victimization (scoring a ‘0’ on the standardized victimization scale or scoring > 1 *SD* above the mean of victimization). For severely victimized youth (> 2 *SD* above mean of victimization) the model negatively predicted perceived cooperation. They were thus *worse off* in classrooms with higher defending descriptive norms (Hypothesis 1).

**Fig. 2:**
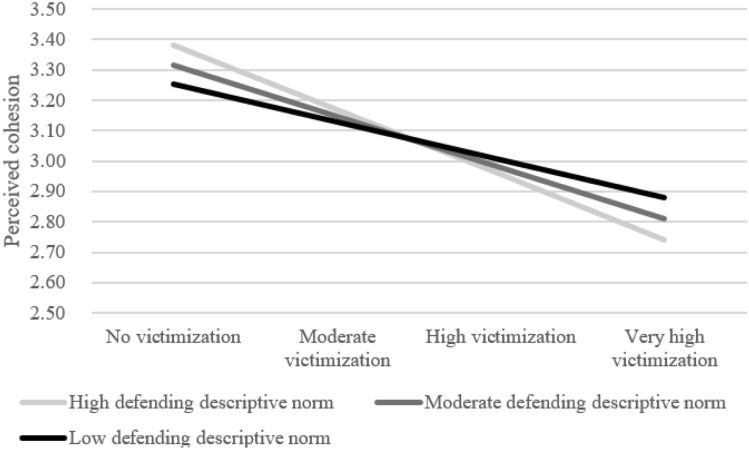
The role of defending descriptive norms in the link between victimization and perceptions of cohesion within classrooms. High defending norms are > 1 SD above the mean whereas low defending norms are > 1 SD under the mean. Low victimization = score of -1 on standardized victimization scale. Moderate victimization = score of 0, high victimization = a score of 1, and very high victimization = 2

The random slope of victimization on perceptions of cohesion was significantly predicted by defending descriptive norms, *B* = -0.04, *SE* = 0.02, *p* = 0.028, explaining 33.3% of the variation in this link between classrooms (Model 3.C.1, Appendix 1). Figure 2 shows that the negative association between victimization and perceived cohesion was stronger when defending descriptive norms were higher. Whereas descriptive norms were positively related to non-victimized youths’ perceptions of cohesion, these norms did *not* seem to matter for youth who experienced moderate or high victimization. Severely victimized youth were even worse off and perceived lower cohesion in classrooms with higher defending descriptive norms (Hypothesis 1).

**Fig. 3:**
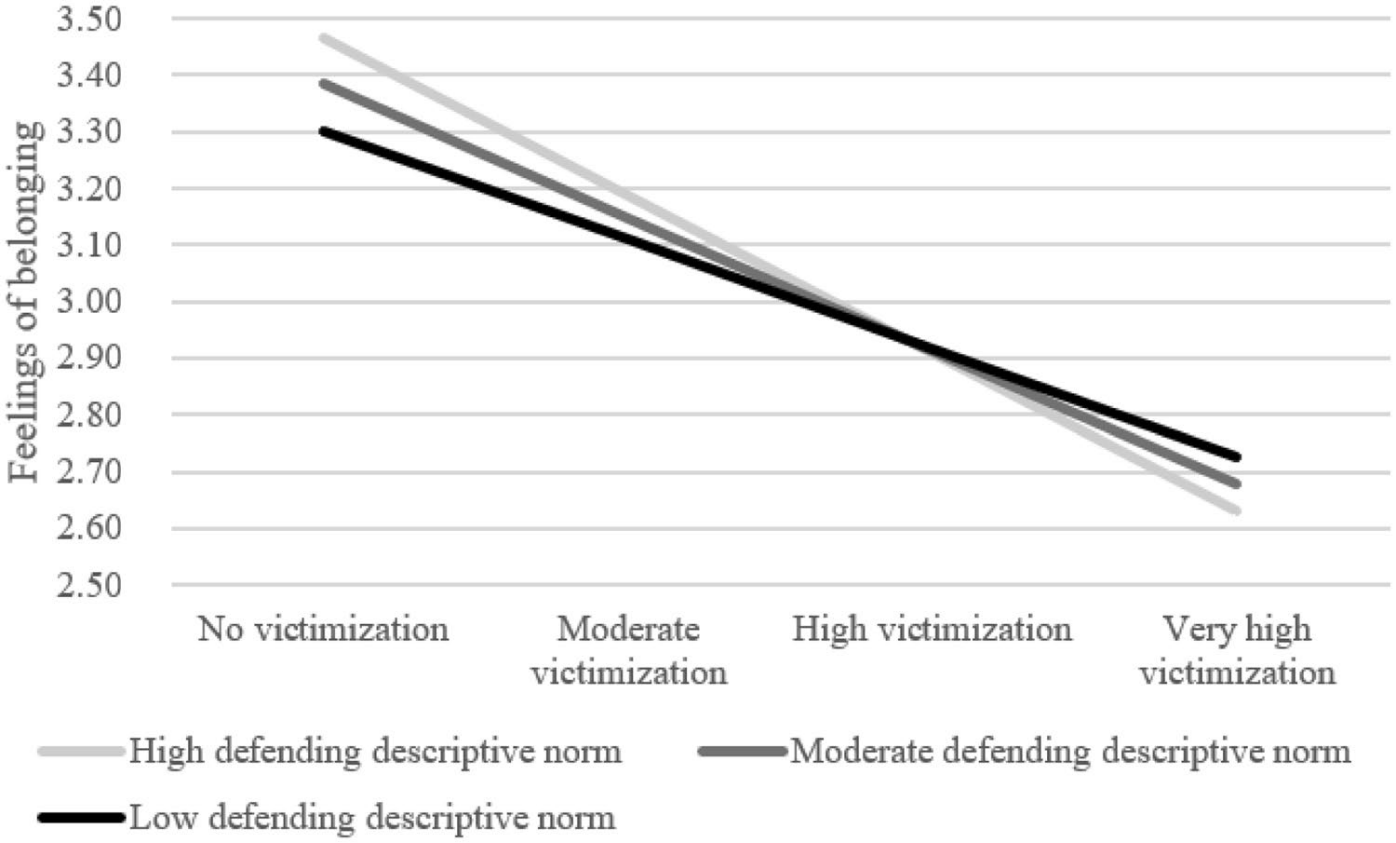
The role of defending descriptive norms in the link between victimization and feelings of belonging within classrooms. High defending norms are > 1 SD above the mean whereas low defending norms are > 1 SD under the mean. Low victimization = score of -1 on standardized victimization scale. Moderate victimization = score of 0, high victimization = a score of 1, and very high victimization = 2

The random slope of victimization on feelings of belonging was significantly predicted by defending descriptive norms, *B* = -0.04, *SE* = 0.02, *p* = 0.033, explaining 40% of the variance between classrooms (Model 5.C.1, Appendix 1). Figure 3 shows that non-victimized youth reported higher feelings of belonging in classrooms with high defending descriptive norms than non-victimized youth in classrooms with low descriptive norms. This link was reversed for youth with severe levels of victimization: they were predicted to have lower feelings of belonging in classrooms with higher defending descriptive norms.

For conflict, isolation, loneliness, and self-esteem there were no significant cross-level interactions with descriptive norms. Findings for these outcomes did not support Hypothesis 1.

### Popularity Defending Norms and Victims’ Adjustment and Classroom Perceptions

There were almost no significant cross-level interactions with victimization and popularity defending norms for any of the outcome variables, indicating that the link between victimization and classroom climate perceptions and adjustment did not vary as a function of the defending popularity norm. The positive *B*-coefficients of popularity norms on classroom perceptions (Tables 2A and 3A) in combination with the non-significant interactions seem to be mostly in line with Hypothesis 2B that popularity norms are *not* susceptible to a healthy context paradox. There was one exception. Popularity norms significantly predicted the random slope of the link between victimization and self-esteem, *B* = -0.10, *SE* = 0.04, *p* = 0.018, explaining 20.0% of the variance between classrooms (Model 6.C.1, Appendix 1). More severely victimized youth reported lower self-esteem, particularly in classrooms with stronger defending popularity norms. This aligns with Hypothesis 2A that victims in classrooms with high defending popularity norms are worse off than victims in classrooms with low defending popularity norms. Because this was the only significant cross-level interaction of seven tested, it should be interpreted with caution. A figure depicting this interaction can be requested by the first author.

### Classroom-level Victimization and Victims’ Adjustment and Classroom Perceptions

No significant cross-level interactions were found for classroom-level victimization explaining the link between individual-level victimization and classroom climate perceptions and psychosocial adjustment. This is in contrast to Hypothesis 3 which stated that victims would be worse off in classrooms with lower levels of victimization.

### Additional Exploratory Analyses: Interactions between Classroom Factors

We conducted exploratory analyses to estimate the interplay of classroom-level factors. We refer to Appendix 2 for a description of the analytic strategy for testing these interactions. The inclusion of extra interaction terms did not change the main findings presented above.

For all outcomes, either main models (Appendix 3, Models A.2) or models with two-way cross-level interactions were preferred (Appendix 3, Models C.2), in a similar pattern as the presented analyses *without* extra interaction variables. There was one exception: for perceived conflict, the model containing three-way cross-level interactions had a better fit (lower AIC) and one significant three-way cross-level interaction. The significant three-way cross-level interaction was between defending popularity norms and classroom-levels of victimization, in the link between individual-level victimization and perceived conflict (*B* = -0.16, SE = 0.06, *p* = 0.004). This interaction indicated that victims reported less conflict in classrooms with strong popularity defending norms, particularly when classroom levels of victimization were low, explaining 60% of the variation in the slope, indicating a strong effect.

There were also significant two-way *classroom-level interactions* (Appendix 3, Model A.2). For feelings of belonging, perceived conflict, and cooperation, the two-way classroom-level interactions between popularity norms and classroom-level of victimization were significant, *B*_feelingsofbelonging_ = 0.16*, SE* = 0.06, *p* = 0.005; *B*_conflict_ = 0.23*, SE* = 0.08, *p* = 0.002; *B*_cooperation_ = 0.19, *SE* = 0.08, *p* = 0.020. The interactions consistently indicated that in classrooms with high levels of victimization, defending popularity norms had advantageous effects by increasing students’ feelings of belonging and perceptions of cooperation, and decreasing their perceptions of conflict. Also, the two-way classroom-level interaction between descriptive norms and classroom-level of victimization was significant for perceived cohesion. It indicated that students perceived more cohesion in the classroom if defending descriptive norms were higher and classroom-levels of victimization were lower (*B* = -0.12, *SE* = 0.03, *p* < 0.001). None of the interactions between descriptive and popularity norms were significant.

### Sensitivity Analyses: Peer-Nominated Victimization

We conducted sensitivity analyses to examine whether findings presented in Tables 2 and 3 were comparable when using peer-nominated rather than self-reported victimization as predicting factor. Students were asked to indicate whom in their classrooms were being bullied. For each student, the number of incoming nominations on this item was counted and divided by the number of potential nominators in the classroom. This measure was significantly, moderately correlated with self-reported victimization (*r* = 0.29). In total, 16.5% of students were mentioned by at least one of their classmates as victim. Findings of analyses with peer-reported victimization were remarkably similar to those with self-reported victimization. Rather similar cross-level interaction effects were detected for descriptive norms and popularity norms. Moreover, descriptive norms were again unrelated to classroom climate perceptions and adjustment, whereas popularity norms did relate to these outcomes. Only two main effects of popularity norms became non-significant, for conflict and isolation. All other findings were highly comparable to those retrieved with self-reported victimization (Appendix 4 and 5).

## Discussion

Recent work has shown that the adjustment difficulties of victimized youth may be exacerbated in healthier classrooms (e.g., classrooms with *low* victimization rates; Huitsing et al. 2012; or *low* aggressive descriptive norms; Morrow et al. 2019). The current study examined whether the healthy context paradox phenomenon also emerges when classrooms are characterized by *positive* aspects that are specifically aimed at making victims to feel better (Cohen and Wills 1985), namely the defending norms. Moreover, we examined whether descriptive and popularity norms would be equally susceptible to this paradox. Findings for descriptive norms were mostly in line with the healthy context paradox: adolescents who experienced higher levels of victimization were *worse off* in classrooms with stronger defending descriptive norms, as they experienced less cooperation, cohesion, and feelings of belonging. This was particularly true for students who were severely victimized. Defending popularity norms did not seem susceptible for the healthy context paradox. Stronger defending popularity norms related to more positive classroom climate perceptions and higher feelings of belonging in general. The impact of defending popularity norms was also true for youth reporting higher levels of victimization (i.e., there were no random slopes of victimization and no cross-level interactions). Thus, defending descriptive norms seem to matter for victims specifically, whereas popularity norms seem to positively affect the classroom as a whole.

### Defending Descriptive Norms

Although stress-buffering theories state that being defended may buffer against victims’ psychosocial maladjustment (Cohen and Wills 1985), the current study indicated that defending descriptive norms did *not* buffer against victims’ problems, and – in line with the healthy context paradox – even increased them. In line with Hypothesis 1, defending descriptive norms negatively affected the link between victimization and perceptions of cohesion and cooperation, and feelings of belonging to the classroom. A potential explanation for why victimized youth may feel less positive about their classroom is that widespread efforts that are made to end bullying are either not effective for them – or they may feel to be just not a part of these efforts, which may enhance negative classroom climate perceptions. It is also possible that victims in classrooms with high defending descriptive norms attribute their victimization to themselves (Schacter et al. 2015), making them feel worse about themselves in relation to their classmates (lower feelings of belonging). Future longitudinal studies are encouraged to examine whether victims’ social comparisons or cognitive attributions are underlying mechanisms explaining why victims may be worse off in more positive classrooms.

In contrast to Hypothesis 1, defending descriptive norms did *not* play a role in victims’ perceptions of conflict and isolation. It could be that in some classrooms, defending norms may co-occur with bullying norms (Laninga-Wijnen et al. 2020b), for instance because high levels of bullying trigger stronger defense responses among bystanders. Thus, it may be that the *combination* of defending and bullying norms rather affects the extent to which (victimized) students experience conflict and isolation in their classroom. Defending descriptive norms did not impact the link between victimization and self-esteem or feelings of loneliness either. It could be that the effect of victimization on maladjustment was so strong, that the context could not add much. Indeed, classroom-level variance was very low for these outcomes, which may have suppressed significant effects. It could also be that the effect of defending norms on victims’ self-esteem and loneliness depends on *how* they are defended and *by whom*. Previous work found that when victimized girls received support from friends who were also victimized, their internalizing problems were exacerbated, perhaps because of co-rumination (Schacter and Juvonen 2020). In the current data, no information was available on who defends whom in what way, making it a valuable direction for future longitudinal studies.

### Defending Popularity Norms

In general, higher defending popularity norms related to higher feelings of belonging and more positive classroom climate perceptions for all students (classroom-level main effects), hence, also for victims (no cross-level interactions). Thus, all students, including victims, benefitted from classroom defending popularity norms. This finding is mostly in line with Hypothesis 2B that popularity norms do not generate the same healthy context paradox as defending descriptive norms. It is likely that in these classrooms, positive and prosocial behaviors such as defending are reputationally salient. That is, they may be seen as valuable for improving one’s popularity. The salience of these behaviors may make students see their classroom positively. Moreover, the visibility and power of popular defenders may clearly signal that bullying is *not* tolerated in these classrooms. A recent study demonstrated that victims are worse off in classrooms high on victim-oriented defending, whereas they were better off in classrooms high on bully-oriented defending (Yun and Juvonen 2020). Youths who prioritize popularity are more likely to engage in bully-oriented defending (Pronk et al. 2019); hence, high defending popularity norms may imply that bullies are publicly confronted, which signals more effectively that bullying is not tolerated. As a result, all students may develop the expectation that – should they face bullying– they can count on their classmates’ help. This fosters positive classroom climate perceptions and feelings of belonging. Future longitudinal studies should test whether the role of defending popularity norms in the classroom climate can indeed be explained by students’ expectations and trust that they can count on each other.

### Interplay of Classroom-Level Factors

Exploratory analyses generated additional insight in how defending norms may interact with victimization in affecting classroom climate perceptions and feelings of belonging. Defending popularity norms seemed particularly fruitful in classrooms with higher levels of victimization, indicating that the norms of popular peers can be an important target for interventions aimed at fostering more positive classrooms. This may be particularly true for classrooms that are in need of such intervention (e.g., with high levels of victimization).

There were no interactions between defending descriptive and popularity norms. This aligns with prior work showing that these two types of norms are empirically distinct (Dijkstra and Gest 2015) and do not interact in affecting peer relationships (Laninga-Wijnen et al. 2019).

## Strengths, Limitations and Future Directions

The current study has several strengths. First, whereas prior work mainly focused on how defending norms affect defenders*,* this study examined how these norms relate to victims’ adjustment. We need to know whether victims are helped by defending norms, as this is a primary aim of anti-bullying programs (Huitsing et al. 2019; Paluck et al. 2016). Second, we extended prior work on the healthy context paradox that, so far, mainly focused on how the absence of negative classroom aspects enhances the plight of victims. Future studies should further examine combinations of positive and negative classroom aspects. Prior work showed that prosocial and aggressive norms may co-occur within some classrooms (Laninga-Wijnen et al. 2020b). In such contexts, aggressive popularity norms overruled the positive ones by fostering aggressive friendships and behaviors, despite prosocial norms. In a similar way, the simultaneous presence of aggressive popularity norms may mitigate potential positive role of defending popularity norms in victims’ adjustment. Third, we demonstrated the importance of distinguishing between descriptive and popularity norms. Descriptive defending norms generated a healthy context paradox, whereas popularity defending norms promoted feelings of belonging and positive classroom perceptions among all students, including victims.

This study also had some limitations. First, we used cross-sectional data. Direction of effects therefore remain unknown. For instance, it could also be that students who do not belong to the classroom, are at particular risk of becoming victimized over time because they lack “social protection” (Hodges and Perry 1999). Longitudinal data could provide more insight in the temporal precedence of these aspects. Moreover, longitudinal data can provide insights in the theoretical explanations that are provided in the current manuscript as potential underlying reasons on *why* the link between victimization and adjustment may vary as a function of the classroom norm (e.g., self-blame, defending inflation). For instance, in a longitudinal study it can be tested whether the effect of being defended depends on whether it contributes to the ceasing of bullying over time. Person-centered analyses may provide insights in whether victims who are strongly being defended, but for whom the bullying does not stop, develop (more) severe feelings of hopelessness and consequently are worse off compared to victims who are not defended at all. Or whether severely victimized youth are particular kind of adolescents, who for instance have depressive predispositions and who regard the world in a negative and distrustful way, and consequently perceive defending attempts of others in a similar way. Moreover, it can be tested whether norms make it more likely that being defended works out adversely or not for such types of victims. The current study provided an important first step, by showing how defending norms work out concurrently for those who are being victimized despite these norms and forms the basis for future longitudinal studies on this new topic.

Second, defending was measured with peer nominations. Not everyone in the classroom may be aware of who is being bullied and who is defending these victims, and whether this is compatible with how victims experience it (Malamut et al. 2020). Moreover, we did not distinguish types of defending. Direct defending (confronting the bully) and indirect defending (consoling the victim) can work out differently for students’ classroom climate perceptions and psychosocial adjustment. For instance, when defenders take a public stance against bullying, it is more likely to affect students’ classroom climate perceptions than when defenders privately comfort victims. Recently, a reliable and valid scale for measuring various of defending was developed (Lambe et al. 2020), which will be valuable to investigate the impact of being defended on victim’s adjustment.

Third, we based our conclusions on seven analyses, for each outcome separately, which may enhance the chance of a Type-I error. At the same time, it should be noted that we tested a complex model on a sample with relatively few cases on the class-level (*n* = 45 classrooms), which limits statistical power to detect significant effects. Further, results were consistently in the same direction, also emerged when examining peer-nominated victimization (sensitivity analyses), and were in line with previous work on the healthy context paradox. Three out of the seven tested cross-level interactions for descriptive norms were significant and explained a relatively large part of the variance in the outcome variable (≥ 20.0%). Moreover, the complete models explained 10.5% to 31.3% of the variance across outcomes, which is comparable to or even larger than other work examining the healthy context paradox. For instance, in a study that conducted longitudinal multi-level regression models to examine the role of the KIVA intervention in victims’ depressive symptoms, social anxiety, self-esteem, and school wellbeing, 2.4% to 10.2% of the variance was explained in these outcomes (Huitsing et al. 2019). In another study that used multi-level regression analyses to examine the role of aggressive norms in the link between victimization and children’s self-views, about 0.5% to 9.8% of the variance was explained at the individual-level (Morrow et al. 2019). The relatively large variance explained in the current study strengthens our confidence that defending norms play a role in some aspects of victims’ adjustment – yet importantly, not in all aspects. We also tested seven interaction effects for classroom levels of victimization and popularity norms respectively. Only one of these was significant, which we did not further interpret to prevent over-interpretation. Replication studies are encouraged to examine whether our found pattern is also consistent across other (longitudinal) studies with larger samples.

Fourth, though investigating the moderating role of norms in the victimization-adjustment link provides important information about contextual differences in victims’ experiences, no insights are provided in more subtle, relational processes. That is, the role of defending on victims’ adjustment may depend on who defends whom in a particular context (Huitsing et al. 2014). For instance, victimized girls who were defended by victimized friends were found to experience increased internalizing problems whereas this was not the case for boys (Schacter and Juvonen 2020). Future studies are encouraged to dig into these questions, in order to better understand whether the effects of being defended on victims’ adjustment within a particular context are dependent on who defends whom.

## Conclusions and Implications

In this study the association between victimization and maladjustment depended on the broader classroom context. In line with the healthy context paradox (Huitsing et al. 2019), victims in classrooms with defending descriptive norms were worse off than victims in classrooms without these norms. Defending popularity norms worked out positively for the general classroom climate perceptions and feelings of belonging of all students, including victims. Therefore, interventions may benefit more from encouraging defending popularity norms than from stimulating defending descriptive norms. Some interventions, such as Meaningful Roles Intervention (Ellis et al. 2016) and the Roots Intervention (Paluck et al. 2016) aim at encouraging defending popularity norms by rewarding prosocial behavior, for example by assigning prosocial leaders in a classroom (who are often popular students) and exchanging compliment cards. Our study provides preliminary evidence for the effectiveness of such interventions. To conclude, this study indicates that defending descriptive norms relate adversely to victims’ classroom climate perceptions and feelings of belongings, whereas defending popularity norms foster positive classroom environments for all students.

## Electronic supplementary material

Below is the link to the electronic supplementary material.

Supplementary file1: **Appendices** (DOCX 27 KB)

## Data Availability

Data and syntaxes will be made available in the online system DANS Easy (Radboud University Nijmegen, The Netherlands) once this paper is published.

## Availability Code

Available upon request by first author
