# Supplementary material for: The Role of Defending Norms in Victims’ Classroom Climate Perceptions and Psychosocial Maladjustment in Secondary School
Source: Res Child Adolesc Psychopathol. 2020 Dec 10;49(2):169–84. doi: 10.1007/s10802-020-00738-0 (PMC7826303; doi:10.1007/s10802-020-00738-0)
Supplement: Supplementary file 1 — Supplementary file1: Appendices (DOCX 27 KB) [file 10802_2020_738_MOESM1_ESM.docx]

Appendices

Appendix 1.

*Comparisons of main models, models including random slope for level of victimization, and models including cross-level interactions.*

| Model comparisons |  | A.1  Main Model | B.1  A.1 + Random slope | C.1  B.1 + Two-way cross-level interactions |
| --- | --- | --- | --- | --- |
| Free parameters |  | 19 | 21 | 24 |
| Classroom Climate |  |  |  |  |
| M.1 Cooperation |  |  |  |  |
| AIC |  | 1828.44 | 1826.14 | 1826.73 |
| BIC |  | 1879.39 | 1887.29 | 1903.16 |
| u1_vic_ |  |  | 0.005 | 0.004 |
| M.2 Conflict |  |  |  |  |
| AIC |  | 1886.10 | 1881.76 | 1883.47 |
| BIC |  | 1937.05 | 1942.90 | 1959.89 |
| u1_vic_ |  |  | 0.005 | 0.004 |
| M.3 Cohesion |  |  |  |  |
| AIC |  | 2307.72 | 2307.57 | 2306.73 |
| BIC |  | 2358.67 | 2368.71 | 2383.15 |
| u1_vic_ |  |  | .003 | .002 |
| M.4 Isolation |  |  |  |  |
| AIC |  | 2187.066 | 2188.59 | 2192.33 |
| BIC |  | 2238.017 | 2249.73 | 2268.75 |
| u1_vic_ |  |  | 0.004 | 0.003 |
| School adjustment |  |  |  |  |
| M.5 Belonging |  |  |  |  |
| AIC |  | 2137.74 | 2134.74 | 2136.03 |
| BIC |  | 2188.69 | 2195.88 | 2212.45 |
| u1_vic_ |  |  | 0.005* | 0.003 |
| M. 6 Self-esteem |  |  |  |  |
| AIC |  | 1551.61 | 1546.56 | 1550.00 |
| BIC |  | 1602.56 | 1607.70 | 1626.43 |
| u1_vic_ |  |  | 0.005 | 0.004 |
| M.7 Loneliness^#^ |  |  |  |  |
| AIC |  | 1421.40 | 1423.61 | 1427.01 |
| BIC |  | 1472.35 | 1484.75 | 1503.43 |
| u1_vic_ |  |  | 0.069 | .069 |

*Note.* # this model has been conducted using logistic multi-level analyses.

Appendix 2.

*Analytic strategy for additional analyses exploring two-way classroom-level interactions and three-way cross-level interactions.*

We explored the interactive effects between classroom-level victimization, defending popularity norms and defending descriptive norms. In a first step (Model A.2, Appendix 2), we ran main models including three two-way classroom-level interactions (defending popularity norm*defending descriptive norm; defending popularity norm*classroom victimization; defending descriptive norm*classroom victimization). In the next model, we added the random slope of victimization on outcomes (Model B.2, Appendix 2). As a third step, the two-way cross-level interactions were added (e.g., defending popularity norm*individual victimization; Model C.2, Appendix 2), and as a fourth step, three-way cross-level interactions (e.g., defending descriptive norm*defending popularity norm*individual victimization) were included. In case of improved AIC and significance of cross-level interactions, cross-level interactions were discussed.

Appendix 3.

*Extension Models on Appendix 1. Comparisons of main models, models including random slope for level of victimization, and models including two-way and three-way cross-level interactions.*

| Model comparisons |  | A.2  A.1 + extra classroom-level interactions | B.2  A.2 + Random slope | C.2  B.2 + Two-way cross-level interactions | D.2  C.2 + Three-way cross-level interactions |
| --- | --- | --- | --- | --- | --- |
| Free parameters |  | 22 | 24 | 27 | 30 |
| Classroom Climate |  |  |  |  |  |
| M.1 Cooperation |  |  |  |  |  |
| AIC |  | 1828.54 | 1825.45 | 1825.20 | 1829.50 |
| BIC |  | 1894.78 | 1901.87 | 1916.91 | 1936.50 |
| u1_vic_ |  |  | 0.005 | 0.004 | 0.004 |
| M.2 Conflict |  |  |  |  |  |
| AIC |  | 1883.46 | 1879.66 | 1881.59 | 1882.17 |
| BIC |  | 1949.69 | 1956.09 | 1973.30 | 1989.16 |
| u1_vic_ |  |  | 0.006 | 0.004 | .002 |
| M.3 Cohesion |  |  |  |  |  |
| AIC |  | 2305.71 | 2306.50 | 2303.93 | 2308.52 |
| BIC |  | 2371.95 | 2382.93 | 2395.639 | 2415.52 |
| u1_vic_ |  |  | .0028 | .0033 | .0034 |
| M.4 Isolation |  |  |  |  |  |
| AIC |  | 2190.83 | 2191.97 | 2195.92 | 2197.44 |
| BIC |  | 2257.06 | 2268.40 | 2287.63 | 2304.43 |
| u1_vic_ |  |  | 0.004 | 0.003 | .001 |
| School adjustment |  |  |  |  |  |
| M.5 Belonging |  |  |  |  |  |
| AIC |  | 2137.07 | 2134.80 | 2134.64 | 2139.72 |
| BIC |  | 2203.31 | 2211.23 | 2226.35 | 2246.72 |
| u1_vic_ |  |  | 0.005* | 0.004 | 0.004 |
| M. 6 Self-esteem |  |  |  |  |  |
| AIC |  | 1556.15 | 1550.99 | 1554.49 | 1560.26 |
| BIC |  | 1622.39 | 1627.41 | 1646.20 | 1667.25 |
| u1_vic_ |  |  | 0.005 | 0.004 | .004 |
| M.7 Loneliness^#^ |  |  |  |  |  |
| AIC |  | 1702.57 | 1704.49 | 1708.14 | 1712.11 |
| BIC |  | 1814.67 | 1826.77 | 1845.71 | 1864.96 |
| u1_vic_ |  |  | 0.080 | .067 | .061 |

| Appendix 4.  *Main models and models with cross-level interactions predicting students’ perceptions of cooperation, conflict, isolation, and cohesion within the classroom, with peer-reported victimization* | | | | | | | | |
| --- | --- | --- | --- | --- | --- | --- | --- | --- |
|  | Cohesion |  | Cooperation |  | Conflict |  | Isolation |  |
|  | A. Main models | B. Interactions | A. Main models | B. Interactions | A. Main models | B. Interactions | A. Main models | B. Interactions |
| *Individual-level predictors* |  |  |  |  |  |  |  |  |
| Gender | -.02(.04) | -.02(.04) | -.01(.03) | -.01(.04) | .13(.04)*** | .12(.03)*** | -.04(.04) | -.05(.04) |
| Age | .02(.04) | .02(.04) | -.02(.04) | -.02(.04) | .00(.04) | .00(.04) | .06(.04) | .06(.04) |
| Victimization | -.09(.06) | -.11(.05)* | -.21(.05)*** | -.23(.09)* | -.03(.07) | -.04(.06) | .12(.06)* | .14(.06)* |
| *Classroom-level predictors* |  |  |  |  |  |  |  |  |
| Grade | .00(.04) | .01(.04) | -.04(.03) | -.01(.05) | .05(.04) | .06(.04)^+^ | .01(.04) | .01(.04) |
| Defending descriptive norm | .04(.04) | .04(.04) | .06(.03) | .06(.03) | .03(.04) | .04(.04) | .03(.04) | .03(.04) |
| Defending popularity norm | .26(.10)** | .26(.10)** | .17(.08)* | .17(.08)* | .19(.12)^+^ | .19(.12) | .18(.14) | .18(.14) |
| Classroom-level victimization | -.99(.38)** | -.99(.37)** | -.96(.27)*** | -.95(.26)** | -1.31(.32)*** | -1.29(.32)*** | -.91(.42)* | -.90(.42)* |
| *Two-way cross-level interactions* |  |  |  |  |  |  |  |  |
| Victimization*descriptive norm | - | -.18(.04)*** | - | -.09(.04)** | - | -.02(.04) | - | -.02(.05) |
| Victimization*popularity norm | - | .02(.15) | - | -.24(.12) | - | -.16(.14) | - | -.22(.14) |
| Victimization*classroom-level victimization | - | .10(.47) | - | -.01(.36) | - | -.04(.72) | - | -.75(.51) |
| *Residual variances* |  |  |  |  |  |  |  |  |
| Residual variance within | .39(.02)*** | .39(.02)*** | .26(.02)*** | .26(.02)*** | .33(.02)*** | .33(.02)*** | .36(.02)*** | .35(.02)*** |
| Residual variance between | .03(.01)*** | .03(.01)** | .02(.01)** | .02(.01)** | .04(.01)* | .04(.02)** | .04(.01)*** | .04(.01)*** |
| *Variance explained* |  |  |  |  |  |  |  |  |
| Variance explained within | .00(.00) |  | .02(.01)* |  | .01(.01)^+^ |  | .01(.01) |  |
| Variance explained between | .48(.14)*** |  | .54(.13)*** |  | .51(.16)** |  | .30(.16)^+^ |  |

| Appendix 5. *Main models and models with cross-level interactions predicting students’ self-esteem, loneliness, and feelings of belonging, with peer-reported victimization* | | | | | | |
| --- | --- | --- | --- | --- | --- | --- |
|  | Self-esteem |  | Loneliness |  | Feelings of belonging |  |
|  | A. Main models | B. Interactions | A. Main models | B. Interactions | A. Main models | B. Interactions |
| *Individual-level predictors* |  |  |  |  |  |  |
| Gender | -.26(.03)*** | -.26(.03)*** | .65(.15)*** | .66(.15)*** | -.15(.04)*** | -.14(.04)*** |
| Age | .01(.03) | .01(.03) | .03(.15) | .02(.15) | -.03(.05) | .-04(.05) |
| Victimization | -.12(.04)** | -.13(.05)** | 1.38(.16)*** | 1.47(.18)* | -.41(.06)*** | -.42(.06)*** |
| *Classroom-level predictors* |  |  |  |  |  |  |
| Grade | .04(.02)* | .04(.02)* | .06(.09) | .06(.10) | -.07(.06) | -.08(.06) |
| Defending descriptive norm | .04(.02)* | .04(.02)* | .00(.11) | -.02(.11) | .05(.03) | .05(.03) |
| Defending popularity norm | .03(.06) | .02(.06) | -.12(.35) | -.19(.35) | .16(.08)* | .17(.08)* |
| Classroom-level victimization | .13(.22) | .14(.22) | 1.07(.34) | 1.08(.15) | -.28(.16) | -.28(.16) |
| *Two-way cross-level interactions* |  |  |  |  |  |  |
| Victimization*descriptive norm | - | .01(.03) | - | .20(.15) | - | -.10(.05)* |
| Victimization*popularity norm | - | -.21(.10)* | - | .81(.40)* | - | -.12(.19) |
| Victimization*classroom-level victimization | - | -.07(.44) | - | -.67(.66) | - | -.02(.44) |
| *Residual variances* |  |  |  |  |  |  |
| Residual variance within | .23(.01)*** | .23(.01)*** | n.a. | n.a. | .36(.02)*** | .35(.02)*** |
| Residual variance between | .003(.002) | .003(.002) | .07(06) | .06(.06) | .01(.01)* | .01(.01)* |
| *Variance explained* |  |  |  |  |  |  |
| Variance explained within | .07(.01)*** |  | .08(.02)*** |  | .06(.03)* |  |
| Variance explained between | .37(.29) |  | .20(.24) |  | .60(.18)*** |  |
